# Chemoprotection of murine hematopoietic cells by combined gene transfer of cytidine deaminase (CDD) and multidrug resistance 1 gene (MDR1)

**DOI:** 10.1186/s13046-015-0260-4

**Published:** 2015-12-12

**Authors:** Sebastian Brennig, Nico Lachmann, Theresa Buchegger, Miriam Hetzel, Axel Schambach, Thomas Moritz

**Affiliations:** Reprogramming and Gene Therapy Group, REBIRTH Cluster-of Excellence, Hannover Medical School, Carl-Neuberg-Str.1, Hannover, D-30625 Germany; Institute of Experimental Hematology, Hannover Medical School, Hannover, Germany; JRG Translational Hematology of Congenital Diseases, REBIRTH Cluster-of Excellence, Hannover Medical School, Hannover, Germany; Division of Hematology/Oncology, Boston Children’s Hospital, Harvard Medical School, Boston, USA

**Keywords:** Chemoprotection, Myeloprotection, Cytidine deaminase, Multidrug resistance 1 gene, Lentiviral vectors, Hematopoietic cells

## Abstract

**Background:**

Hematologic toxicity represents a major side effect of cytotoxic chemotherapy frequently preventing adequately dosed chemotherapy application and impeding therapeutic success. Transgenic (over)expression of chemotherapy resistance (CTX-R) genes in hematopoietic stem- and progenitor cells represents a potential strategy to overcome this problem. To apply this concept in the context of acute myeloid leukemia and myelodysplasia, we have investigated the overexpression of the multidrug resistance 1 (MDR1) and the cytidine deaminase (CDD) gene conferring resistance to anthracyclines and cytarabine (Ara-C), the two most important drugs in the treatment of these diseases.

**Methods:**

State-of-the-art, third generation, self-inactivating (SIN) lentiviral vectors were utilized to overexpress a human CDD-cDNA and a codon-optimized human MDR1-cDNA corrected for cryptic splice sites from a spleen focus forming virus derived internal promoter. Studies were performed in myeloid 32D cells as well as primary lineage marker negative (lin^−^) murine bone marrow cells and flow cytometric analysis of suspension cultures and clonogenic analysis of vector transduced cells following cytotoxic drug challenge were utilized as read outs.

**Results:**

Efficient chemoprotection of CDD and MDR1 transduced hematopoietic 32D as well as primary lin^−^ cells was proven in the context of Ara-C and anthracycline application. Both, CTX-R transduced 32D as well as primary hematopoietic cells displayed marked resistance at concentrations 5–20 times the LD_50_ of non-transduced control cells. Moreover, simultaneous CDD/MDR1 gene transfer resulted in similar protection levels even when combined Ara-C anthracycline treatment was applied. Furthermore, significant enrichment of transduced cells was observed upon cytotoxic drug administration.

**Conclusions:**

Our data demonstrate efficient chemoprotection as well as enrichment of transduced cells in hematopoietic cell lines as well as primary murine hematopoietic progenitor cells following Ara-C and/or anthracycline application, arguing for the efficacy as well as feasibility of our approach and warranting further evaluation of this concept.

**Electronic supplementary material:**

The online version of this article (doi:10.1186/s13046-015-0260-4) contains supplementary material, which is available to authorized users.

## Background

Cytotoxic chemotherapy represents a major component of anti-cancer treatment strategies. However, cytotoxic agents not only affect the malignant target cells, but also are associated with substantial damage to healthy cells and tissues. In particular hematologic toxicity represents a major and frequently dose-limiting side effect that may cause severe infectious complications and thrombocytopenias, prohibit further application of chemotherapy on time, and impede therapeutic success. Transgenic (over)expression of chemotherapy resistance (CTX-R) genes in hematopoietic stem- and progenitor cells, also referred to as chemo- or myeloprotective gene therapy, represents a potential strategy to overcome this problem [1, 2]. In this regard, a number of CTX-R genes have been identified and studied for their myeloprotective properties [1]. Of note, recently effective protection from alkylating agent-induced myelotoxicity has been demonstrated following gene transfer of the CTX-R gene mutant O^6^-methylguanine methyltransferase (mutMGMT) in a cohort of glioblastoma patients treated with temozolomide [3, 4]. In this study, profound in vivo enrichment of genetically modified hematopoietic cells and clinical benefits in comparison to a disease matched control group was demonstrated, establishing a proof-of-concept for the clinical applicability and efficacy of myeloprotective gene therapy approaches. Other myeloprotective strategies interfere with antimetabolite type cytotoxic drugs. This includes mutant forms of dihydrofolate reductase (mutDHFR), protecting cells from antifolate drugs such as methotrexate and trimetrexate [5–7] as well as knock-down of hypoxanthine phosphoribosyl transferase (HPRT) expression protecting cells from purine analogs like 6-thioguanine [8].

Multi-drug resistance 1 (MDR1) and cytidine deaminase (CDD) represent two other well-studied CTX-R genes. The human MDR1 gene encodes the cellular efflux protein ABCB1 (P-glycoprotein, P-gp), a member of the ATP-binding cassette superfamily, which confers resistance to a wide variety of clinically relevant chemotherapeutic agents including anthracyclines, epipodophyllotoxins, taxoids or vinca-alkaloids [9]. Transgenic expression of MDR1 by γ-retroviral vectors protects murine and human hematopoietic cells from the toxic effects of paclitaxel, vincristine, etoposide, doxorubicin or daunorubicin *in vitro* [10–15]. Moreover, for various of these agents in vivo protection of murine and human hematopoietic cells has been demonstrated following γ-retroviral gene-transfer to HSCs in murine [10, 16–18] and humanized (NOD/SCID) [19] transplant models, respectively, and chemoprotection of human hematopoietic progenitor cells *in vitro* also has been reported following lentiviral mediated gene transfer of MDR1 [20, 21]. Furthermore, MDR1 has been successfully used as a selection marker during hematopoietic stem cell gene therapy (HSC-GT) [18, 22]. Despite these pre-clinical achievements early clinical trials with MDR1 in the late 1990s showed only moderate success primarily due to low gene transfer efficacy [23–27] or aberrant splicing of the MDR1 gene [28]. CDD codes for an enzyme of the nucleotide salvage pathway and protects cells against such clinically relevant agents as cytosine-arabinoside (Ara-C), gemcitabine, decitabine and azacytidine [29]. Meanwhile, CDD-mediated drug resistance and enrichment of transduced cells following γ-retroviral gene transfer has been established in murine and human hematopoietic cells *in vitro* [30–32] as well as murine long-term reconstituting hematopoietic stem cells (HSC) [33, 34]. Though, a potential lymphotoxicity of CDD overexpression was noted in one of these studies [34], this problem was circumvented when doxycycline-induced transgene expression from a lentiviral vector backbone was employed [35].

Combinations of Ara-C and anthracyclines as in the classical “3 + 7” or TAD regimen [36] are highly effective in the treatment of acute myeloid leukemia or high-risk myelodysplasia and represent the backbone of chemotherapy in these disease entities. However, these regimens are associated with a profound and long-lasting myelosuppression. This may create problems particularly in relapsed disease situations and/or in the elderly, where an already compromised hematopoietic stem cell compartment even aggravates these side effects. To overcome this dilemma hematopoietic stem cell gene therapy allowing for the combined overexpression of CDD and MDR1 appears as a logical strategy to protect the lymphohematopoietic system from combined chemotherapy. Thus, we here have evaluated this concept applying state-of-the-art lentiviral gene transfer technology. Our data demonstrate highly efficient chemoprotection as well as enrichment of transduced cells in hematopoietic cell lines as well as primary murine hematopoietic progenitor cells following combined Ara-C/anthracycline application.

## Methods

### Lentiviral vector constructs and preparations

Lentiviral vectors were based on 3^rd^ generation SIN lentiviral vectors modified with a woodchuck hepatitis virus-derived xposttranscriptional-regulatory element [37, 38]. RRL.PPT.SFFV.hMDR1.IRES.GFPpre* (referred to as LV.SFFV.MDR1) contained a human codon-optimized multidrug resistance gene 1 (*hMDR1*) cDNA additionally modified by removing (cryptic) splice donor and acceptor sites to increase tRNA usage and thereby increase expression levels. The *hMDR1*-cDNA was inserted with AgeI/SalI restriction enzymes into RRL.PPT.SFFV.pre* construct. Subsequently, an enhanced green fluorescent protein (eGFP) was inserted together with an IRES site via SalI. RRL.PPT.SFFV.hCDD.IRES.GFP.pre* and RRL.PPT.SFFV.hCDD.IRES.dtomato.pre* (referred to as LV.SFFV.CDD) were generated by cloning of the cDNA of human cytidine deaminase (*hCDD*) (former OpenBiosystems, IHS1380-OB-97652440, Epsom, UK) via AgeI/SalI followed by SalI mediated insertion of an IRES site linked to a green (GFP) or red (dtomato) fluorescent protein. RRL.PPT.SFFV.hCDD.P2A.hMDR1.IRES.GFP.pre* (referred to as LV.SFFV.CDD.2A.MDR1) was generated by insertion of *hCDD* and *hMDR1* and a porcine teschovirus-1 (P2A) linker sequence using overlap/extension PCR. Subsequently, the *hCDD* fragment was inserted into LV.SFFV.MDR1 by AgeI followed by introduction of IRES.GFP via SalI. RRL.PPT.SFFV.GFP.pre* (referred to as LV.SFFV.GFP) was cloned by insertion of GFP reporter into RRL.PPT.SFFV.pre*. Production as well as titration was performed as previously described [39]. Titers (TU/mL) ranged from 2×10^6^ - 9×10^6^ for LV.SFFV.MDR1, 1×10^6^ - 1×10^7^ for LV.SFFV.CDD.2A.MDR1, 3×10^7^ - 2×10^8^ for LV.SFFV.CDD, and 2×10^7^ - 2×10^8^ for LV.SFFV.GFP control vector.

### Experiments with 32D myeloid cells

#### Culture

Murine 32D cells were cultured in RPMI-1640 supplemented with 10 % fetal calf serum (Biochrom, Berlin, Germany), 100 U/ml penicillin/streptomycin (Pen/Strep), 2 mmol/l glutamine (all Life Technologies) and 2 ng/ml mIL-3 (Peprotech, Hamburg, Germany).

#### Transduction

Genetic modification of 32D cells was performed by adding viral supernatant to cells in the presence of 10 μg/ml protaminsulfate (Carl Roth, Karlsruhe, Germany) at 37 °C. Twenty-four hours after transduction, cells were washed, expanded for several days and subsequently sorted for fluorescent reporter gene expression (FACS AriaIIu, Becton Dickinson) to establish transgenic 32D cells of purity ≥ 90 %.

#### *In vitro* protection

Chemoprotection was carried out by seeding 1.5×10^5^ cells in 2 ml complete medium and cytotoxic drugs were added in given concentrations. Following three days incubation, cell survival was analyzed by flow cytometry (using FSC/SSC exclusion). Stock solutions of cytotoxic drugs were prepared by central pharmacy of Hannover Medical School.

#### Transgene expression analysis

To analyze expression of *hMDR1*, RNA was isolated from cells previously treated with cytotoxic drugs as well as non-treated controls using TRIsure reagent (Bioline, London, UK) as recommended by the manufacturer. Following DNase I digestion, RNA was reverse transcribed using RevertAid reverse transcriptase and oligo(dT) primers (all Thermo Scientific, Schwerte, Germany). Subsequently, 25 ng cDNA was used for quantification by SYBR Green-based qRT-PCR on a StepOnePlus Real-Time PCR System (Thermo Scientific, Schwerte, Germany Biosystems). Expression of *hDMR1* mRNA is given relative to non-treated untransduced control cells (normalized to endogenous murine β-actin). Expression of hCDD protein was analyzed by Western Blot as described previously [40] using cells previously treated with cytotoxic drugs as well as non-treated controls.

#### *In vitro* proliferation assay

Cell proliferation analysis of (transduced) 32D cells was determined using cell proliferation dye eFluor 670 (eBioscience, San Diego, CA, USA). In brief, cells were incubated with the proliferation dye as recommended by the manufacturer and analyzed for initial dye uptake using flow cytometry (FL4 channel, FACS Calibur, Becton Dickinson). Subsequently, cells were applied to an *in vitro* protection assay as described above and three days later analyzed by flow cytometry.

### Experiments with primary murine hematopoietic cells

#### Isolation

Bone marrow of C57BL/6 (Janvier Laboratories, Saint Berthevin Cedex, France and Central animal facility, Hannover Medical School, Hannover, Germany) mice was harvested from femora and tibiae and lineage negative (lin^−^) cells were purified using MACS separation (Lineage Cell depletion kit, Miltenyi, Bergisch Gladbach, Germany). Cells were cultured and prestimulated for 24 h in StemSpan medium (StemCell Technologies, Cologne, Germany) supplemented with 10 ng/ml rmSCF, 20 ng/ml rmTPO, 20 ng/ml rmIGF and 10 ng/ml rhFGF (all PeproTech) prior to transduction.

#### Transduction

Transduction of lin^−^ cells with lentiviral vectors was carried out using retronection (10 mg/cm^2^; Takara, Otsu, Japan) -coated dishes as recommended by the manufacturer. Approximately three to four days later, non-sorted cells were applied to myeloid *in vitro* differentiation experiments or cells were sorted for GFP reporter gene expression and subjected to a clonogenic progenitor assays.

#### Clonogenic progenitor assays

Clonogenic growth of hematopoietic progenitor cells were assessed by incubating 1.500 transduced lin- cells previously sorted for eGFP expression. Clonogenic cultures were performed in 1 ml IMDM/1.3 % methylcellulose supplemented with 15 % fetal calf serum, 2 % bovine serum albumin, 2 mM L-glutamine, 50 μM 2-Mercaptoethanol, 10 μg/ml rh-insulin, 200 μg/ml human transferrin, 50 ng/ml rm-SCF, 10 ng/ml rm-IL3, 10 ng/ml rm-IL6 and 5 IU/ml rh-EPO (HSC007, R&D Systems, Wiesbaden-Nordenstadt, Germany) in the presence of different concentrations of cytotoxic drugs. Colonies of more than 50 cells were counted after 6–7 days.

#### Myeloid *in vitro* differentiation

*In vitro* protection and selection was performed using 5×10^4^ non-sorted transduced lin^−^ cells. Cells were cultured in RPMI-1640 supplemented with 10 % fetal calf serum, 100 U/ml penicillin/streptomycin (Pen/Strep), 2 mmol/l glutamine and addition of 20 ng/ml rmIL-3 and 100 ng/mL rh-G-CSF (Peprotech, Hamburg, Germany). Cells were treated with cytotoxic drugs in the given concentration for three days and analyzed by flow cytometry. For *in vitro* selection, data are given as fold increase in % GFP^+^ cells with non-treated cells = 1.

#### Statistical analysis

All graphs were created using Prism V5 (GraphPad) and statistical analysis was performed using Prism V6 software (GraphPad, La Jolla, CA, USA). Unless otherwise noted analysis of variance (ANOVA) with recommended post hoc testing was performed.

## Results

### Generation of lentiviral vectors

**Fig. 1:**
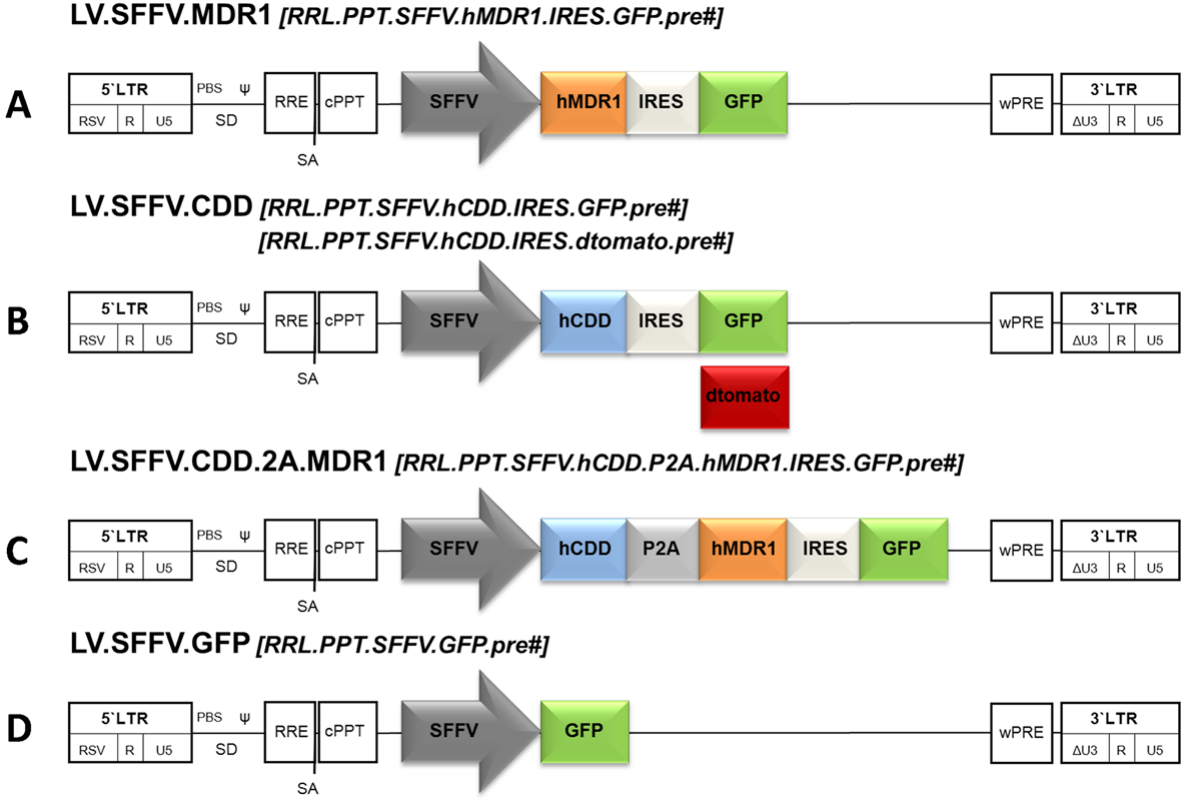
Lentiviral vectors (LV) for transgenic MDR1 and CDD expression. 3^rd^ generation self-inactivating (SIN) lentiviral vectors containing (**a**) codon-optimized human MDR1-cDNA, (**b**) human CDD-cDNA, (**c**) human CDD-cDNA linked via porcine teschovirus-1 (P2A) sequence to human codon-optimized MDR1 or (**d**) enhanced green fluorescent protein (GFP)-cDNA. All vectors carry a spleen focus forming virus (SFFV) promoter and bicistronic vectors in A-C contain an internal ribosomal entry site (IRES) followed by green- or red fluorescent protein cDNA (GFP and dtomato). LV vectors contain 5′ and 3′ long terminal repeats with SIN deletion in the U3 region (LTRs, ΔU3, R, U5), splice donor (SD) and splice acceptor (SA) sites, the post-transcriptional regulatory element of woodchuck hepatitis virus (wPRE), a central polypurine tract (cPPT), the Rev responsive element (RRE) and an extended encapsidation signal (Ψ) including the 5′ region of gag (ΔGA)

To express CTX-R genes in hematopoietic target cells, third generation SIN lentiviral vectors were generated. Lentiviral constructs were equipped with a codon-optimized version of the human multidrug resistance 1 gene (*hMDR1*) cDNA additionally corrected for cryptic splice sites (LV.SFFV.MDR1) or a cDNA encoding for human cytidine deaminase (*hCDD;* LV.SFFV.CDD) used in combination with the fluorescence markers GFP or dTomato. Simultaneous expression of *hCDD* and *hMDR1* in hematopoietic cells was accomplished either by co-transduction with two vectors (only in 32D cells) or by transduction with a vector containing *hCDD* and *hMDR1* linked via a porcine teschovirus-1 (P2A) linker sequence (LV.SFFV.CDD.2A.MDR1). In all constructs transgene expression was driven by an internal spleen focus forming virus (SFFV) promoter, and a vector solely expressing GFP served as a control (Fig. 1).

### Efficient protection of 32D cells from cytotoxic drug treatment

**Fig. 2:**
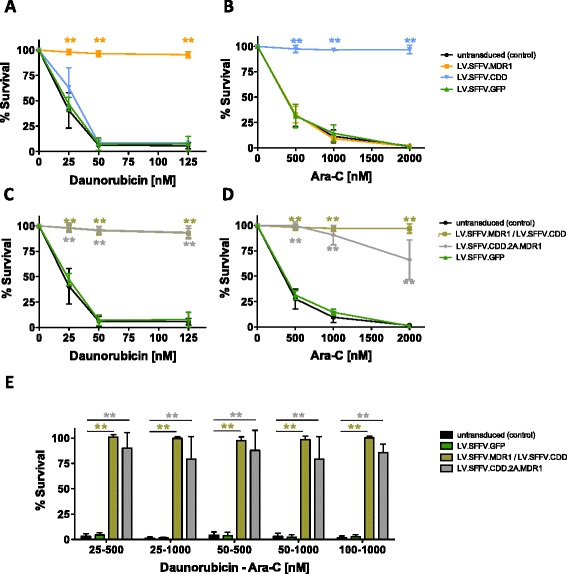
Chemoprotection of murine 32D cells following transgenic expression of MDR1 and CDD. 32D cells transduced with LV.SFFV.MDR1, LV.SFFV.CDD or LV.SFFV.GFP lentiviral vectors were treated with (**a**) daunorubicin (*n* = 3–11) or (**b**) Ara-C (*n* = 4–10) monotherapy. 32D cells genetically modified with two drug resistance genes either by co-transduction of LV.SFFV.MDR1 and LV.SFFV.CDD or by LV.SFFV.CDD.2A.MDR1 transduction as well as LV.SFFV.GFP transduced cells were treated with (**c**) daunorubicin (*n* = 3–11), (**d**) Ara-C (*n* = 4–11) monotherapy or (**e**) combined daunorubicin/Ara-C therapy (*n* = 3–12). Data are presented as mean ± SD; *p ≤ 0.05/**p ≤ 0.01 denote significant differences compared to untransduced control (calculated by ANOVA)

To prove functionality of our lentiviral vectors, initial experiments were carried out in murine myeloid 32D cells. Median transduction rates of 32D cells as determined by reporter expression were 16 % for LV.SFFV.MDR1, 6.7 % for LV.CDD.2A.MDR1, 43.5 % for LV.SFFV.CDD and 84 % for LV.SFFV.GFP. Following transduction cells were enriched by fluorescence-activated cell sorting (FACS) based on reporter gene expression to achieve populations with ≥ 92 % purity. MDR1 and CDD transgene expression in these populations were confirmed by qRT-PCR or Western blot, respectively (Additional file 1: Figure S1). Subsequently, cells were treated with chemotherapeutic agents and first experiments were performed with vectors carrying a single CTX-R gene. Here LV.SFFV.MDR1 gene-modified cells displayed a classical multi-drug resistance phenotype demonstrated by significant protection from typical MDR1-associated drugs including the anthracyclines daunorubicin (≥125 nM) and doxorubicin (≥250 nM) (Fig. 2a, Additional file 2: Figure S2A) as well as the taxoid derivative paclitaxel and the epipodophyllotoxin etoposide (Additional file 2: Figure S2B,C). Of note, for all four drugs, transgenic MDR1 expression conferred profound chemoprotection with virtually 100 % cell survival even at concentrations in excess of 5- (anthracyclines, etoposide) or even 40-times (paclitaxel) the LD_50_ of not transduced control cells. As expected, overexpression of CDD using the LV.SFFV.CDD construct did not result in protection against daunorubicin (Fig. 2a), however, when Ara-C instead of daunorubicin was administered, LV.SFFV.CDD gene-modified cells revealed significantly increased chemoresistance (≥2000 nM) and were protected from concentrations more than 5-times the LD_50_ of non-transduced control or MDR1 gene-modified cells (Fig. 2b).

Next, we evaluated the functionality of combined CDD/MDR1 expression in 32D cells. To this point, cells were either co-transduced with the two single CTX-R vectors (LV.SFFV.CDD and LV.SFFV.MDR1) or modified with the LV.SFFV.CDD.2A.MDR1 vector expressing both genes, CDD and MDR1, from a single construct. Again, transgenic cells were enriched by FACS and thereafter treated with daunorubicin or Ara-C. Both LV.SFFV.CDD/LV.SFFV.MDR1 as well as LV.SFFV.CDD.2A.MDR1 gene-modified cells were protected against daunorubicin monotherapy (≥125 nM), whereas control cells were susceptible from 25 nM onwards (Fig. 2c). Similar results were observed when cells were treated with Ara-C, except for a slightly reduced resistance to very high Ara-C concentrations (2000 nM) in LV.SFFV.CDD.2A.MDR1 transduced cells (Fig. 2d). Finally, cells expressing CDD and MDR1 were treated with a combination of daunorubicin and Ara-C. While non-transduced 32D control cells were highly susceptible to all drug combinations applied, LV.SFFV.CDD/LV.SFFV.MDR1 as well as LV.SFFV.CDD.2A.MDR1 gene-modified cells demonstrated marked resistance to the daunorubicin/Ara-C combination with > 80 % of cells surviving even at the highest doses tested. Likewise percentages of surviving cells were significantly increased ranging from 17-100-fold for LV.SFFV.CDD/ LV.SFFV.MDR1 and 19-110-fold for LV.SFFV.CDD.2A.MDR1, respectively (Fig. 2e).

Results of these cytotoxicity studies were confirmed by cell proliferation analysis of e670 labelled 32D cells. In these studies susceptibility of 32D cells to daunorubicin as well as Ara-C was clearly associated with cell cycle arrest, whereas MDR1 or CDD transgene expression reconstituted the proliferative potential of 32D cells also in the presence of the respective drugs (Additional file 3: Figure S3).

Therefore, our data clearly show that expression of CDD and MDR1 alone or in combination results in increased myeloprotection in the context of anthracycline and/or nucleoside-analogue therapy.

### Chemoprotection of primary hematopoietic progenitor cells

Next, we employed our lentiviral vectors in primary murine hematopoietic progenitor cells. To this point, lin^−^ murine bone marrow cells were transduced and following FACS-based enrichment for GFP expression, seeded into clonogenic assays in the presence of cytotoxic drugs. Median transduction rates of primary cells used for clonogenic as well as suspension experiments (s. below) as determined by GFP reporter expression were lower for vectors containing MDR1 (4.6 % and 4.9 % for LV.SFFV.MDR1 and LV.CDD.2A.MDR1, respectively) than for LV.SFFV.CDD (49 %) and LV.SFFV.GFP control (50 %).

**Fig. 3:**
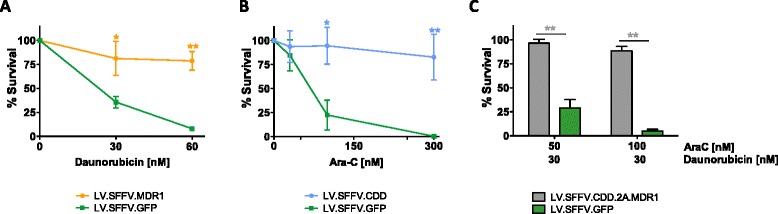
Chemoprotection of primary murine hematopoietic progenitor cells genetically engineered with MDR1 and CDD. Gene-modified lin^−^ hematopoietic progenitor cells were applied to a clonogenic progenitor assay. **a** LV.SFFV.MDR1 as well as LV.SFFV.GFP gene-modified cells were treated with daunorubicin monotherapy (*n* = 3), (**b**) LV.SFFV.CDD as well as LV.SFFV.GFP transduced cells were treated with Ara-C monotherapy (*n* = 3) and (**c**) LV.SFFV.CDD.2A.MDR1 as well as LV.SFFV.GFP gene-modified cells were treated with daunorubicin/Ara-C combination therapy (*n* = 4). Data are presented as mean ± SEM; *p ≤ 0.05/** p ≤ 0.01 denote significant differences compared to LV.SFFV.GFP control (calculated by ANOVA)

First, cells transduced with LV.SFFV.MDR1 were analyzed. As evident from Fig. 3a clonogenic growth from these cells clearly was protected from the daunorubicin treatment with virtually unperturbed colony growth observed in the presence of up to 60 nM of the drug. In contrast, colony growth of LV.SFFV.GFP and mock-transduced control cells was significantly reduced already from 30 nM daunorubicin onwards (Fig. 3a, Additional file 4: Figure S4A). Similarly, survival of LV.SFFV.CDD gene-modified cells was analyzed in the presence of increasing concentrations of Ara-C. Again a profound protection mediated by the CTX-R gene was detected. While clonogenic growth of control cells was markedly reduced at 150 nM and nearly absent at 300 nM of Ara-C, LV.SFFV.CDD transduced cells were completely protected from Ara-C toxicity at both dose levels investigated (Fig. 3b, Additional file 4: Figure S4B). Next, lin^−^ cells were transduced with LV.SFFV.CDD.2A.MDR1 lentiviral vector and colony formation was studied following combined daunorubicin/Ara-C challenge. LV.SFFV.CDD.2A.MDR1 gene-modified cells demonstrated a marked drug resistance compared to LV.SFFV.GFP transduced control cells when treated with a combination of 30 nM daunorubicin and 50 or 100 nM Ara-C. Less than 50 % of surviving colonies were observed in control cells with the 30/50 combination and this was even further reduced when Ara-C doses were increased to 100 nM (Fig. 3c, Additional file 4: Figure S4C). In contrast, more than 86 % of LV.SFFV.CDD.2A.MDR1 gene-modified clonogenic progenitor cells survived at the 30/50 and 78 % at the 30/100 dose level, highlighting the profound protection conferred by the LV.SFFV.CDD.2A.MDR1 vector to hematopoietic progenitors in the context of combined anthracycline/nucleoside analog treatment (Fig. 3c).

**Fig. 4:**
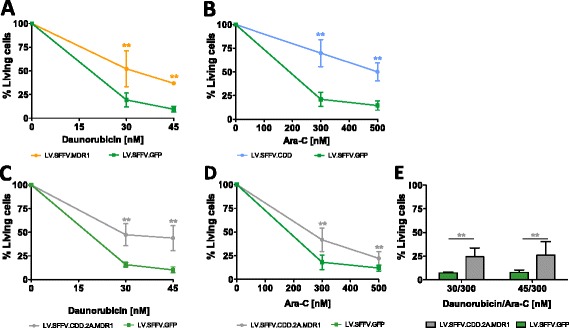
Enhanced survival of CTX-R gene-modified primary murine hematopoietic cells during myeloid differentiation. Non-sorted genetically modified lin^−^ cells were subjected to myeloid differentiation in suspension culture in the presence of cytotoxic drugs. **a** LV.SFFV.MDR1 as well as LV.SFFV.GFP gene-modified cells were treated with daunorubicin monotherapy (*n* = 4–7), and (**b**) LV.SFFV.CDD as well as LV.SFFV.GFP transduced cells were treated with Ara-C monotherapy (*n* = 7). LV.SFFV.CDD.2A.MDR1 as well as LV.SFFV.GFP were treated with (**c**) daunorubicin (*n* = 6), (**d**) Ara-C monotherapy (*n* = 7–12) or (**e**) daunorubicin/Ara-C combination therapy (*n* = 5–6). Data are presented as mean ± SD; *p ≤ 0.05/** p ≤ 0.01 denote significant differences compared to LV.SFFV.GFP control (calculated by ANOVA)

These results were confirmed when transduced lin^−^ cells were exposed to the cytotoxic drugs in suspension cultures in the presence of IL-3 and G-CSF for three days. Again LV.SFFV.MDR1 transduced cells were significantly protected from daunorubicin (Fig. 4a, Additional file 4: Figure S4D) and LV.SFFV.CDD gene-modified cells from Ara-C (Fig. 4b, Additional file 4: Figure S4E) while simultaneous expression of MDR1 and CDD from the LV.SFFV.CDD.2A.MDR1 yielded protection from both agents (Fig. 4c, d, Additional file 4: Figure S4F). In comparison to LV.SFFV.GFP transduced control cells, proportions of live cells at the end of the experiment were significantly increased following daunorubicin (2.4-4.2-fold for LV.SFFV.MDR1 and 3-4-fold for LV.SFFV.CDD.2A.MDR1) as well as Ara-C (3.3-fold for LV.SFFV.CDD and 2.2-fold for LV.SFFV.CDD.2A.MDR1) application. Again, genetic modification with the LV.SFFV.CDD.2A.MDR1 vector revealed a significant protection from combined daunorubicin/Ara-C application (Fig. 4e).

### Marked selection of CTX-R gene-modified cells following chemotherapy application

**Fig. 5:**
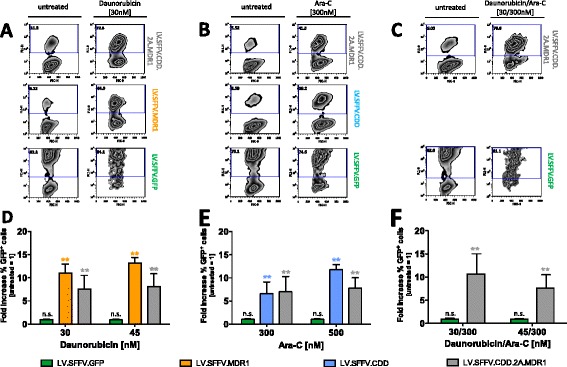
*In vitro* selection of CTX-R gene-modified primary murine hematopoietic cells during myeloid differentiation. Non-sorted genetically modified lin^−^ cells were treated with cytotoxic drugs in mIL-3/h-GCSF supported suspension culture. **a**-**c** Representative flow cytometric data are given to show enrichment of gene-marked cells analyzed by GFP reporter gene expression (FL-1). **d** LV.SFFV.MDR1, LV.SFFV.CDD.2A.MDR1 as well as LV.SFFV.GFP gene-modified cells were treated with daunorubicin monotherapy (*n* = 3–7), and (**e**) LV.SFFV.CDD, LV.SFFV.CDD.2A.MDR1 as well as LV.SFFV.GFP transduced cells were treated with Ara-C monotherapy (*n* = 7–12). **f** LV.SFFV.CDD.2A.MDR1 as well as LV.SFFV.GFP gene-modified cells were treated with daunorubicin/Ara-C combination therapy (*n* = 4–6). Data are presented as fold increase in % GFP^+^ cells (non-treated cells = 1) and mean ± SD are given; *p ≤ 0.05/**p ≤ 0.01 denote significant differences compared to untreated cells with the same vector (calculated by ANOVA)

Notably, considerable *in vitro* enrichment of CTX-R gene-modified cells upon cytotoxic drug treatment was observed in the latter studies. While gene-marking in non-treated lin^−^ cells in cytokine supported suspension cultures ranged from 1 to 9 % for the LV.SFFV.MDR1, and from 5 to 6 % for the LV.SFFV.CDD vector, these were increased to 67-80 % (LV.SFFV.MDR1) and 56-75 % (LV.SFFV.CDD) after culture in the presence of 45 nM daunorubicin and 500 nM Ara-C, respectively (Additional file 5: Table S1). For the LV.SFFV.MDR1 vector this constitutes an 11.0 ± 1.9 and 13.2 ± 1.2 -fold enrichment of transduced cells of in the presence of 30 and 45 nM daunorubicin, respectively (Fig. 5d). Similar results were generated for the LV.SFFV.CDD construct with a 6.5 ± 2.6 and 11.8 ± 1.1 -fold enrichment in the presence of 300 and 500 nM of Ara-C (Fig. 5e).

Marked and significant *in vitro* selection also was observed for the LV.SFFV.CDD.2A.MDR1 construct. While 3-20 % of gene marking were observed following transduction this was increased to 36-84 % respective 23-66 % following exposure to daunorubicin or Ara-C alone (Fig. 5d, e and Additional file 5: Table S1) and to 59-85 % upon combined drug application. This correlated to a 10.7 ± 4.4 (30/300) and 7.7 ± 2.8 -fold (45/300) enrichment upon treatment with the two daunorubicin/Ara-C combinations (Fig. 5f). No enrichment has been observed in LV.SFFV.GFP transduced control cells. Taken together, these data clearly show an enhanced survival and substantial *in vitro* selection of CTX-R gene-modified cells upon cytotoxic drug treatment applied as single agent as well as combination therapy.

## Discussion

Transplantation of *ex vivo* CTX-R gene modified hematopoietic stem-and progenitor cells may reduce cytotoxic drug induced myelotoxicity and allow for dose-intensified or prolonged chemotherapy application. Towards this objective, we have generated lentiviral vectors expressing MDR1 as well as CDD to genetically engineer murine hematopoietic cells for increased inherent resistance to anthracycline- and nucleoside-analogue-type cytotoxic drugs. Utilizing these vectors, we demonstrate highly efficient chemoprotection as well as *in vitro* enrichment of transduced hematopoietic cells in the context of Ara-C as well as anthracycline application. Moreover, and of particular importance, dual expression of the CDD as well as the MDR1 transgene conferred significant cellular resistance to combined nucleoside analogue/anthracycline application.

Transgenic expression of CDD successfully conferred resistance to Ara-C in myeloid 32D as well as primary murine bone marrow cells. The degree of Ara-C resistance achieved was comparable to that described with LTR-driven γ-retroviral vectors in the past [30, 32, 35, 41]. Similarly, resistance to MDR1 associated drugs conferred to 32D and primary hematopoietic cells by transgenic MDR1 (over)expression was in the same range as reported in most studies before [10–12]. On this ground we also would expect similar in vivo efficacy of our constructs as previously demonstrated for –retroviral vectors in murine or humanized transplant models [18, 19, 33, 34]. Most importantly, however, effective protection from combined Ara-C/anthracycline application by the CDD/MDR1 combination was shown in our experiments demonstrating the feasibility to functionally co-express both genes in hematopoietic cells. Given that anti-cancer chemotherapy usually is applied as a combination of multiple agents, simultaneous transfer of CTX-R genes, represents a logical strategy for myeloprotection, and particularly combinations including MDR1 have been studied. Combination partners have been, glutathione S-transferase (GST), MGMT or mutDHFR to allow for additional protection against alkylators such as nitrosoureas, temozolomide (MGMT), chlorambucil, melphalan (GST), as well as folate antagonists [13, 14, 20, 42, 43]. Similarly, CDD has been combined with GST [44] and mutDHFR [45–47] to add nitrogen mustards and folate antagonists to the resistance spectrum. In our attempt to transfer the protection concept to AML and MDS treatment settings, we now for the first time demonstrate the successful combination of CDD and MDR1.

In our study, safety-optimized 3^rd^ generation SIN lentiviral vectors were utilized. While effective myeloprotection was observed for all constructs, transduction efficiency and titers were reduced with vectors containing MDR1, most likely due to the size of the transgene cassette. As this may be problematic for in vivo applications, gene transfer efficacy needs to be improved. Here, addition of transduction enhancers, such as rapamycin or cylosporin A, engineering of the vector surface phenotype, or suitable *ex vivo* selection strategies prior to transplant may increase efficacy [48–51]. Co-expression of MDR1 and CDD was accomplished with co-transduction of cells with single gene vectors (in case of 32D cells) or by using the LV.SFFV.CDD.2A.MDR1 combination vector. In this vector, CDD and MDR1 are linked via a porcine teschovirus-1 (P2A) linker sequence that, in contrast to internal ribosomal entry (IRES) sites, should result in equimolar expression of both transgenes [52, 53]. In our experiments however, LV.SFFV.CDD.2A.MDR1 transduction, though highly effective, resulted in moderately reduced Ara-C resistance compared to double-transduced or LV.SFFV.CDD -transduced cells indicating reduced CDD expression from the LV.SFFV.CDD.2A.MDR1 vector. In this context, position dependency of transgene expression resulting in altered protection levels recently was shown for vectors combining MDR1 and MGMT via an F2A site. In addition, the residual “2A” tag on the upstream CDD protein may negatively influence transgene function [53].

In our study, efficient selection of CTX-R expressing cells was demonstrated following cytotoxic drug administration. This may be of considerable relevance for the clinical translation of our strategy, given the relative low transduction levels achieved with our MDR1 vector constructs so far. Though a myeloproliferative syndrome has been described in mice transplanted with MDR1 gene-modified hematopoietic cells following extensive *ex vivo* expansion, this almost certain was related to the LTR-driven design of the γ-retroviral vector as well as the applied culture conditions utilized in this study and has not been reproduced with modern safety-improved vector constructs [54]. Efficient selection of MDR1 gene-modified cells convincingly has been demonstrated for murine as well as human hematopoietic cells [18, 48, 55] and selection also has been demonstrated for the CDD/Ara-C system *in vitro* [32, 56] and in a murine transplant model [33].

Given the therapeutic efficacy of Ara-C/anthracycline combinations in AML as well as high-risk MDS, combined CDD/MDR1 gene-transfer as a clinical scenario clearly is directed towards these disease entities. Here, consolidation treatment of high-risk disease states (e.g. second remission or even refractory disease) following secondary transplantation can be envisioned as the primary scenario. In these highly unfavorable disease situations, the potential benefits of additional chemotherapy given with few hematologic effects on disease outcome as well as live quality would clearly offset the potential genotoxic risks associated with the procedure. Moreover, an inadvertent transduction of leukemic cells by the CTX-R transgenes would be prevented by the allogeneic setting. If proven effective and safe, myeloprotection by the CDD/ MDR1 combination may have potential to improve disease outcome in the elderly, especially following alloHSCT employing reduced intensity conditioning regimen. Here the application of additional chemotherapy is associated with increased hematotoxicity frequently resulting in profound and long-lasting myelosuppression and severe infectious complication. Due to its primarily hematological side effects, at least at conventional doses, cytarabine in the context of CDD/MDR1 induced myeloprotection may be applied even at enhanced doses [57, 58], while dose-intensification of anthracyclines will be limited by its cardiotoxicity [59].

Certainly safety constitutes a critical aspect also of myeloprotective gene therapy approaches, and insertional mutagenesis clearly represents a concern with the integrating vectors. While the SFFV promoter used in this study provides high level of transgene expression in human HSCs when incorporated into lentiviral vectors [60], it is also associated with a higher genotoxic risk compared to weaker physiological promoters [61]. In this context it is reassuring that a markedly reduced genotoxic potential has been demonstrated for novel generation SIN lentiviral vectors such as the ones used in our studies [62]. Moreover, no genotoxic events such as profound clonal dominance or leukemias have been encountered in the current generation of hematopoietic gene therapy trial employing safety-optimized y-retroviral or lentiviral SIN vectors [63, 64]. This is of particular importance in the setting of myeloprotective gene therapy, since insertional mutagenesis may give rise to drug-resistant leukemic cells. Incorporation of suicide genes such as herpes simplex virus thymidine kinase (HSV-TK) or inducible caspase 9 should be considered as a fail-safe mechanism [65, 66].

## Conclusions

In conclusion, we have demonstrated significant *in vitro* protection and selection of CDD and MDR1 gene-modified hematopoietic cells utilizing a safety-improved 3^rd^ generation lentiviral gene transfer system as a first step towards the concept of myeloprotective gene therapy in the context of AML and MDS treatment. Now, the next step to further provide evidence for the efficacy as well as safety of this approach will require in vivo studies in relevant animal system including treatment studies in murine leukemia models.

## Additional files

Additional file 1: Figure S1. Transgene expression of hMDR1 and hCDD in gene-modified 32D cells. Transgene expression of gene-modified cells was analyzed either before or after three day exposure to daunorubicin [50nM], Ara-C [1000nM] or both cytotoxic drugs (daunorunicin/Ara-C: [50nM/1000nM] combination). **(A)** hMDR1 mRNA expression is shown in LV.SFFV.MDR1 and LV.SFFV.CDD.2A.MDR1 transduced 32D cells (*n* = 1, technical replicates are shown; data are given relative to untransduced (non-treated) control) and **(B)** expression of hCDD protein is shown for LV.SFFV.CDD and LV.SFFV.CDD.2A.MDR1 gene-modified cells (*n* = 1; vinculin used as loading control). (PDF 292 kb) Additional file 2: Figure S2. Multidrug-resistance phenotype of murine MDR1 gene-modified 32D cells. 32D cells transduced with LV.SFFV.MDR1 or LV.SFFV.GFP lentiviral vector, and treated with **(A)** doxorubicin (*n* = 4–5) or **(B)** paclitaxel (*n* = 4–5) or **(C)** etoposide (*n* = 4–5) monotherapy are shown. Data are presented as mean ± SD; *p ≤ 0.05/**p ≤ 0.01 denote significant differences compared to untransduced control (calculated by ANOVA). (PDF 198 kb) Additional file 3: Figure S3. Transgenic expression of CTX-R genes in 32D cells prevents cytotoxic drug-mediated cell cycle arrest. Cell proliferation capacity of untransduced as well as CTX-R gene-modified cells was analyzed by flow cytometric analysis following e670 labelling. The assay was performed in the absence of cytotoxic drugs (upper row) as well as in the presence of daunorubicin (second row), Ara-C (third row) or both drugs (last row). Cells were analyzed three days post treatment (*n* = 1). (PDF 364 kb) Additional file 4: Figure S4. Mock-transduced primary murine hematopoietic cells are susceptible to cytotoxic drug treatment. **(A-C)** Mock-transduced as well as FACS sorted CTX-R gene-modified lin^−^ hematopoietic progenitor cells were seeded in a clonogenic assays in the absence or presence of cytotoxic drugs [*n* = 1; data are given as mean (technical duplicates)]. **(D-F)** Mock-transduced and non-sorted genetically modified lin^−^ cells were treated with cytotoxic drugs in mIL-3/h-GCSF supported suspension culture (*n* = 2–4; data are given as mean ± SD). (PDF 157 kb) Additional file 5: Table S1.
*In vitro* selection of primary hematopoietic gene-modified cells. Data for *in vitro* selection experiments are given as % GFP^+^ cells (three days post treatment). (DOC 28 kb)
