# Supplementary figures and images for: Chemoprotection of murine hematopoietic cells by combined gene transfer of cytidine deaminase (CDD) and multidrug resistance 1 gene (MDR1)
Source: J Exp Clin Cancer Res. 2015 Dec 12;34:148. doi: 10.1186/s13046-015-0260-4 (PMC4676838; doi:10.1186/s13046-015-0260-4)

Supplementary Figure 1

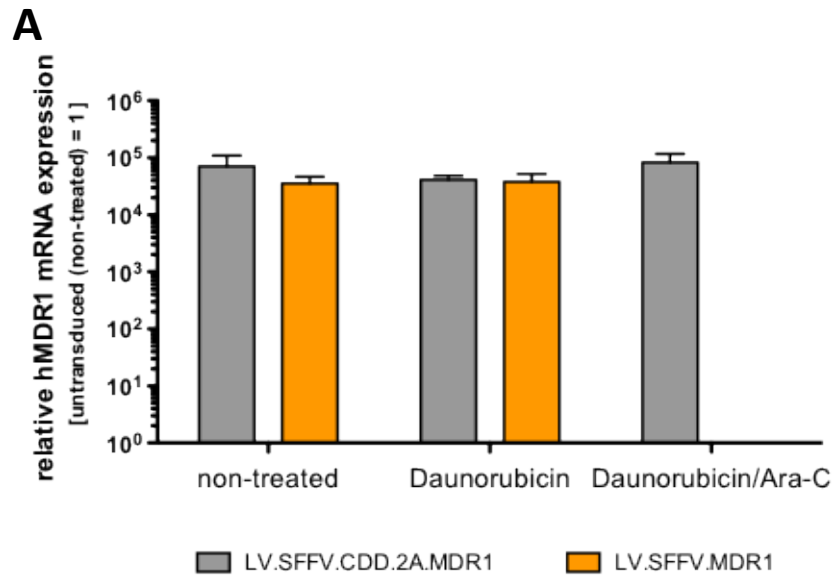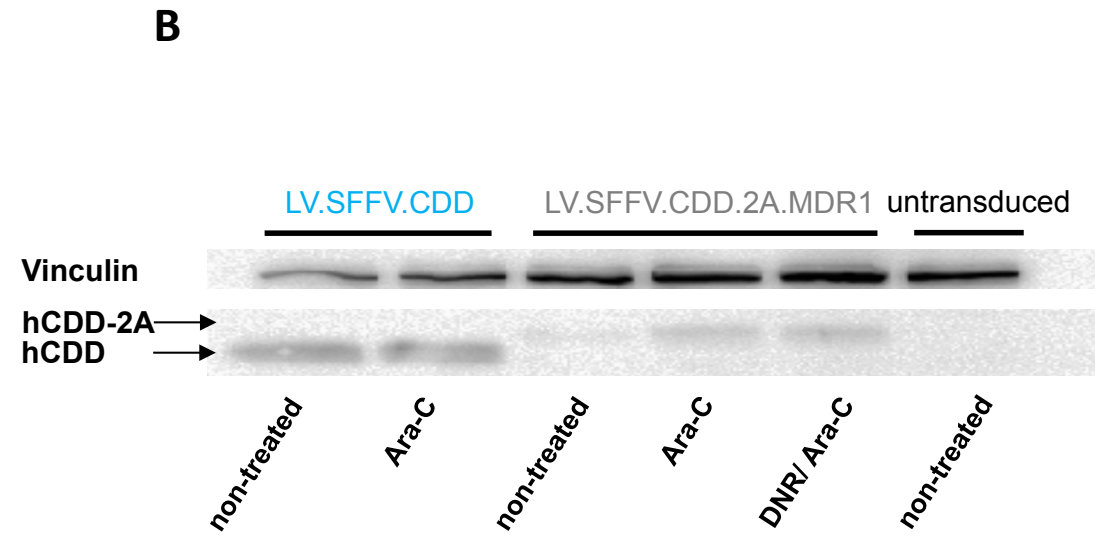

Supplement: Additional file 1: Figure S1. — Transgene expression of hMDR1 and hCDD in gene-modified 32D cells. Transgene expression of gene-modified cells was analyzed either before or after three day exposure to daunorubicin [50nM], Ara-C [1000nM] or both cytotoxic drugs (daunorunicin/Ara-C: [50nM/1000nM] combination). (A) hMDR1 mRNA expression is shown in LV.SFFV.MDR1 and LV.SFFV.CDD.2A.MDR1 transduced 32D cells (n = 1, technical replicates are shown; data are given relative to untransduced (non-treated) control) and (B) expression of hCDD protein is shown for LV.SFFV.CDD and LV.SFFV.CDD.2A.MDR1 gene-modified cells (n = 1; vinculin used as loading control). (PDF 292 kb) [file 13046_2015_260_MOESM1_ESM.pdf]

Supplementary Figure 2

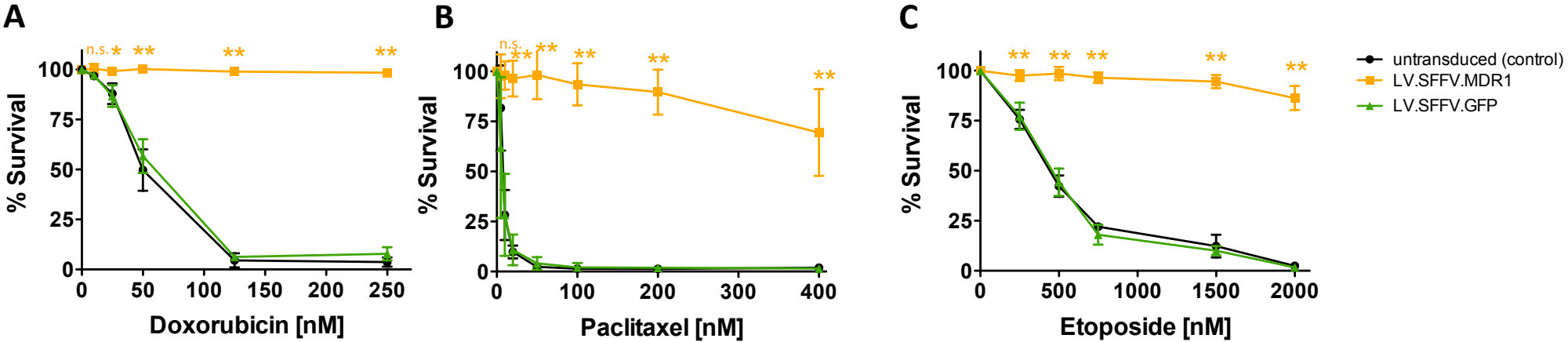

Supplement: Additional file 2: Figure S2. — Multidrug-resistance phenotype of murine MDR1 gene-modified 32D cells. 32D cells transduced with LV.SFFV.MDR1 or LV.SFFV.GFP lentiviral vector, and treated with (A) doxorubicin (n = 4–5) or (B) paclitaxel (n = 4–5) or (C) etoposide (n = 4–5) monotherapy are shown. Data are presented as mean ± SD; *p ≤ 0.05/**p ≤ 0.01 denote significant differences compared to untransduced control (calculated by ANOVA). (PDF 198 kb) [file 13046_2015_260_MOESM2_ESM.pdf]

Supplementary Figure 3

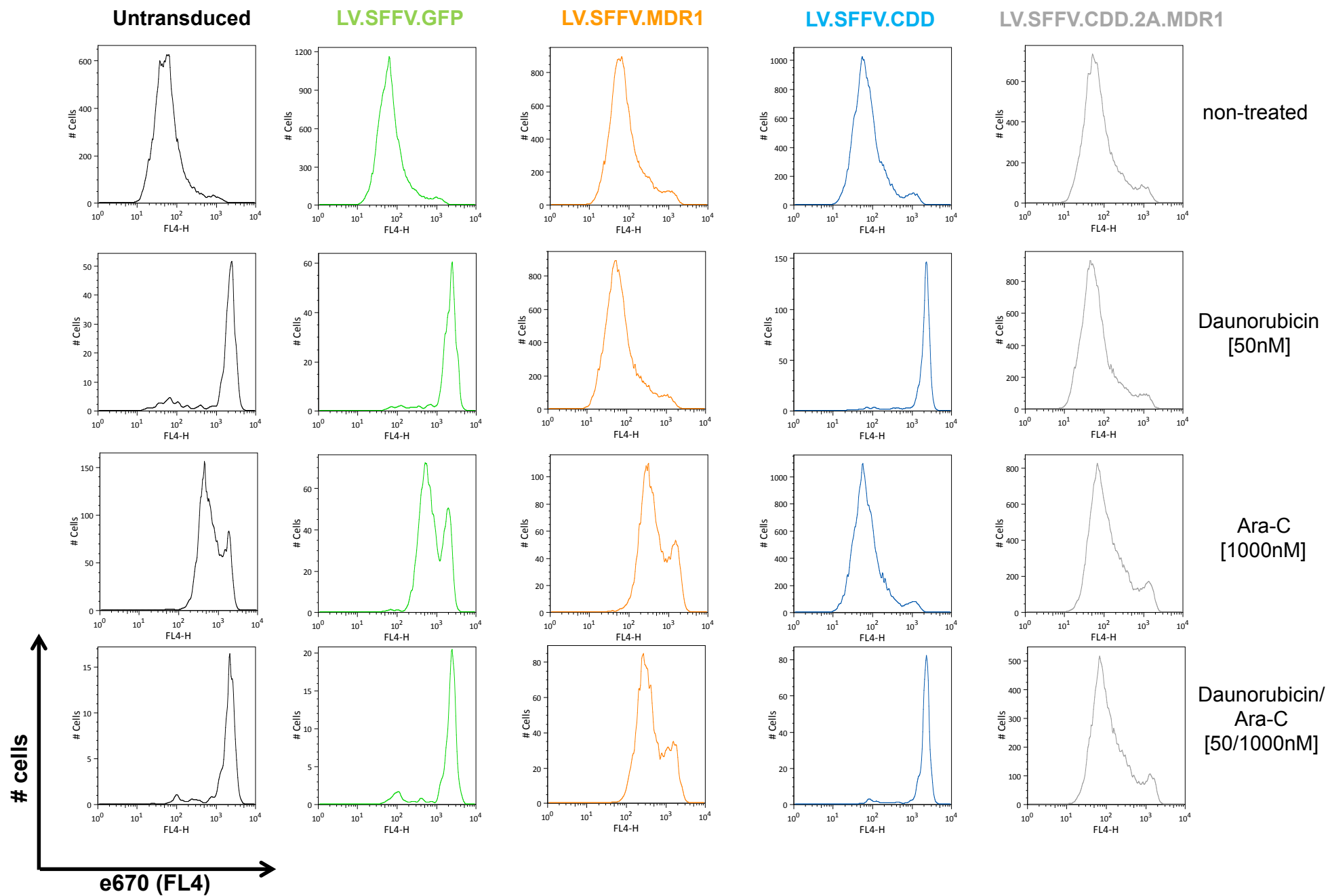

Supplement: Additional file 3: Figure S3. — Transgenic expression of CTX-R genes in 32D cells prevents cytotoxic drug-mediated cell cycle arrest. Cell proliferation capacity of untransduced as well as CTX-R gene-modified cells was analyzed by flow cytometric analysis following e670 labelling. The assay was performed in the absence of cytotoxic drugs (upper row) as well as in the presence of daunorubicin (second row), Ara-C (third row) or both drugs (last row). Cells were analyzed three days post treatment (n = 1). (PDF 364 kb) [file 13046_2015_260_MOESM3_ESM.pdf]

Supplementary Figure 4

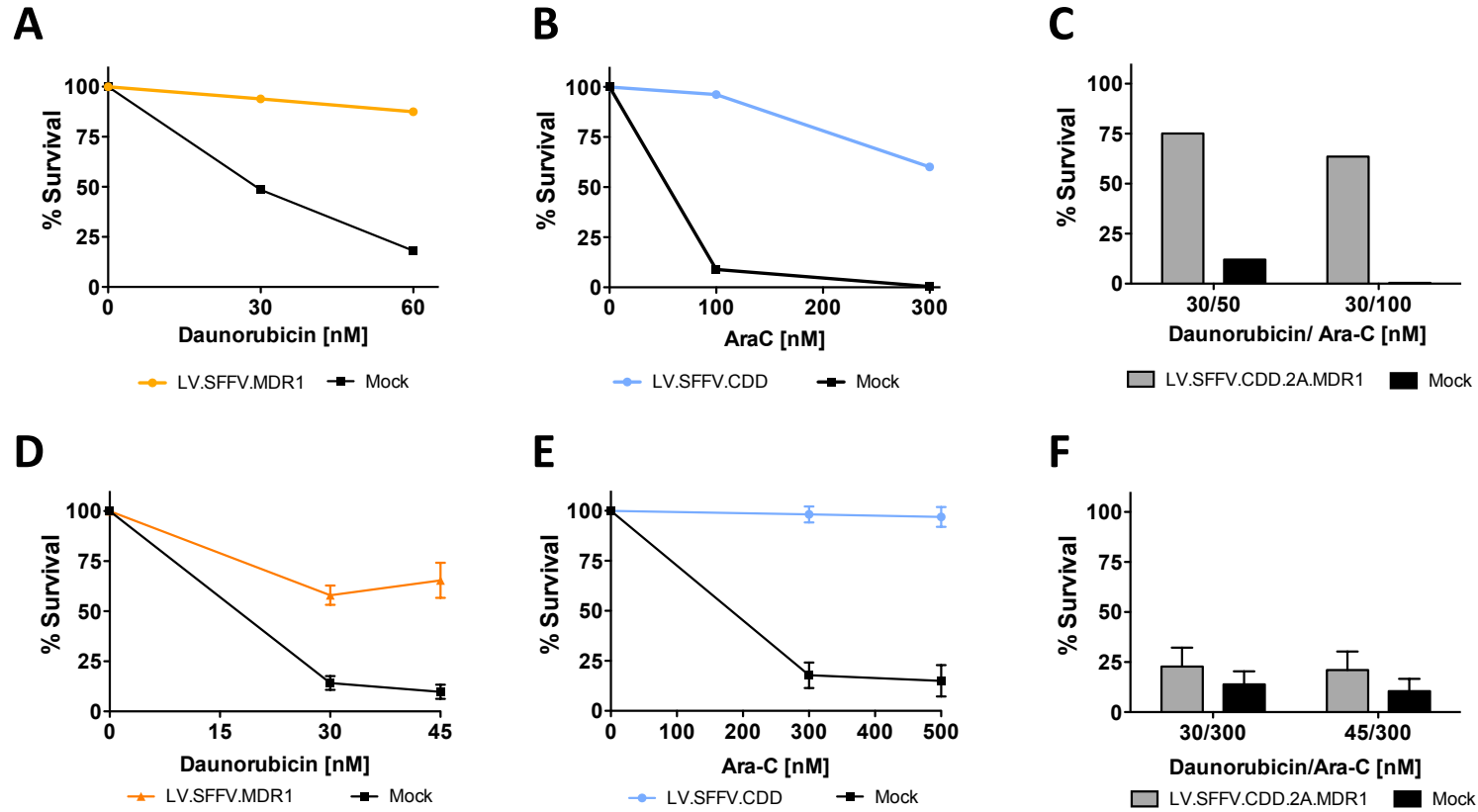

Supplement: Additional file 4: Figure S4. — Mock-transduced primary murine hematopoietic cells are susceptible to cytotoxic drug treatment. (A-C) Mock-transduced as well as FACS sorted CTX-R gene-modified lin− hematopoietic progenitor cells were seeded in a clonogenic assays in the absence or presence of cytotoxic drugs [n = 1; data are given as mean (technical duplicates)]. (D-F) Mock-transduced and non-sorted genetically modified lin− cells were treated with cytotoxic drugs in mIL-3/h-GCSF supported suspension culture (n = 2–4; data are given as mean ± SD). (PDF 157 kb) [file 13046_2015_260_MOESM4_ESM.pdf]
