# Supplementary material for: Chemoprotection of murine hematopoietic cells by combined gene transfer of cytidine deaminase (CDD) and multidrug resistance 1 gene (MDR1)
Source: J Exp Clin Cancer Res. 2015 Dec 12;34:148. doi: 10.1186/s13046-015-0260-4 (PMC4676838; doi:10.1186/s13046-015-0260-4)
Supplement: Additional file 5: Table S1. — In vitro selection of primary hematopoietic gene-modified cells. Data for in vitro selection experiments are given as % GFP+ cells (three days post treatment). (DOC 28 kb) [file 13046_2015_260_MOESM5_ESM.doc]

**Supplementary Table 1:**

**Supplementary table 1: *In vitro* selection of primary hematopoietic gene-modified cells**. Data for *in vitro* selection experiments are given as % GFP^+^ cells (three days post treatment).
